# Tobacco smoking and nicotine delivery alternatives: patterns of product use and perceptions in 13 countries

**DOI:** 10.12688/f1000research.17635.2

**Published:** 2019-05-13

**Authors:** Farhad Riahi, Sarah Rajkumar, Derek Yach

**Affiliations:** 1 Health, Science & Technology, Foundation for a Smoke-Free World, Basel, 4052, Switzerland; 2 Foundation for a Smoke-Free World, New York, NY, 10017, USA

**Keywords:** tobacco, smoking, cigarettes, quitting, nicotine replacement therapy, electronic nicotine delivery systems

## Abstract

**Background:** Smoking tobacco products remains a significant public health problem. The Foundation for a Smoke-Free World commissioned a 13-country survey to gain a clearer understanding of the current landscape of smoking behavior and preferences across the world.

**Methods:** Over 17,000 participants in 13 countries, representing different regions and income groups, answered questions on their smoking patterns and product use, their social context, their motivation to smoke, quit, or switch, and their perception of risks of products and substances. Rim weighting was done for each country to align responses with population demographics, and an additional 200 smokers for each country were surveyed to achieve sufficient sample size for sub-analyses of smoker data.

**Results:** The observed prevalence of smoking ranged from an age-adjusted high of 57.5% in Lebanon to 8.4% in New Zealand among men, with lower rates for women. The majority of smokers were between 25-54 years old, had daily routines and social patterns associated with smoking, used boxed cigarettes, and rated their health more poorly compared to never smokers. Among a range of products and substances, smokers tended to give both cigarettes and nicotine the highest harm ratings. Smokers in high income countries were largely familiar with electronic nicotine delivery systems; the most commonly given reasons for using them were to cut down or quit smoking. A majority of smokers had tried to quit at least once, and while many tried without assistance, motivations, intentions, and methods for smoking cessation, including professional help, nicotine replacement therapies or medications, or electronic cigarettes, varied among countries.

**Conclusions:** Smoking is deeply integrated in smokers’ lives worldwide. Although a majority of smokers have tried to quit, and are concerned for their health, they do not seek help. Smokers lack understanding of the harmful components of smoking tobacco products and the risk profile of alternatives.

## Introduction

While the worldwide rate of tobacco smoking has declined substantially in recent years, the absolute number of people currently smoking has increased from approximately 720 million smokers in 1980 to an estimated 1.1 billion today, the consequence of population growth outpacing declining smoking prevalence in many low and middle income countries (LMICs) ^1– 3^. Additionally, there are significant differences between countries in terms of the epidemiology of smoking and tobacco product preferences.

The ramifications of smoking are well-known. In 2015, smoking was the second leading risk factor for death and disability worldwide and accounted for 11.5% of the world’s deaths and 6.0% of global disability-adjusted life years ^4^. Of the 7.1 million deaths attributed to tobacco use in 2016, 6.3 million were from cigarette smoking ^5^.

The majority of smokers say they want to quit. An analysis of 10 years of National Health Interview Surveys in the United States (US) reported that in 2015, 68% of smokers wanted to quit and 55.4% had tried to quit within the previous year; however, only 7.4% were successful in quitting that year despite a range of available smoking cessation counseling and pharmacologic options ^6^.

In recent years, the use of electronic nicotine delivery systems (ENDS) such as e-cigarettes have gained popularity in many high-income countries, with some evidence that they are being used as tools to reduce or quit smoking ^7^. Although the health effects of these products are still under investigation, some public health experts suggest they may be used as harm reduction products and smoking cessation tools ^8– 10^.

To tackle the enormous global health smoking crisis effectively, more information is needed on the behavior and perceptions of smokers, ex-smokers, and never-smokers towards tobacco products and alternatives. The Foundation for a Smoke-Free World (FSFW) commissioned one of the largest global surveys of smoking habits in order to glean a better understanding of the current landscape of tobacco product use, the population’s grasp of the harm caused by different tobacco products and alternatives, reasons for smoking and for trying to quit or switch, as well as choices of smoking cessation methods.

## Methods

The FSFW commissioned the consulting and research agency Kantar Public to develop and execute a global survey on adult smoking in 13 countries: Brazil, France, Greece, India, Israel, Lebanon, Japan, Malawi, New Zealand, Russia, South Africa, the United Kingdom (UK), and the US. These countries were selected to represent a variety of markets in terms of income level, smoking prevalence, and smoking habits.

Kantar Public developed an 81-question quantitative survey based on existing publications and publicly available data on smoking habits and perceptions. The survey covered four domains: epidemiology of smoking and product use; social context of smokers; motivation to smoke, quit, or switch; and risk perception of products and substances. The survey and sources used are available. Kantar Public upholds the best market research industry practices and all personal data were anonymized so personal data no longer related to identifiable persons and subjects cannot be reidentified. All respondents were volunteers and gave oral (personal interview) or written (online surveys) consent. Oral consent was obtained as some participants may have limited proficiency in reading and writing. Before answering the survey, all respondents received information on what the research was about, what their participation in the project entailed, and any risks involved. Due to the low-risk nature of the study design, the FSFW and Kantar Public did not seek Institutional Review Board approval for the survey.

The survey was piloted with telephone interviews targeting two smokers, two ex-smokers, and two never smokers in each of the 13 countries. Pilot survey respondents were nonrandomly chosen from a contact list based on their smoking status. Smokers were defined as those who responded that they currently smoked cigarettes, cigars, cigarillos or a pipe (or bidis in India) “regularly” or “occasionally;” ex-smokers were defined as those who responded that they used to smoke but stopped; and never smokers were defined as those who responded that they had never smoked.

The sampling plan of the main sample was designed to be nationally representative of all adult citizens (18+) living in the country. Persons below 18 years were excluded from the survey.

In France, Israel, Japan, New Zealand, the UK, and the US, where potential responders in each stratum could be reached via a generic email invitation, respondents completed the survey online in their native language. Participants were stratified according to the most up-to-date census data, with quota definitions based on gender, age, and region to ensure that survey results represented the most accurate estimations of the target populations. Online panels depend on non-probabilistic sampling procedures, in which potential respondents voluntarily sign up to participate in the panel in general and in the survey in particular, which might induce a certain self-selection bias. In order to limit such bias, a large and diverse sampling frame and an effective sampling procedure were set up, sending out only generic survey invitations that did not give any indication of the topic.

Participants in seven countries (Brazil, Greece, India, Lebanon, Malawi, Lebanon, Russia) where email outreach would be inadequate answered the survey face-to-face. The interviewers used validated scripts in the participant’s native language. A stratified random probability sampling approach was used for the interviews. A unit selection performed at each step of the sampling process ensured a completely random approach. Based on the official population statistics, a certain number of primary sampling units (PSUs) were selected randomly, covering both urban and rural areas. According to the overall target sample, the number of interviews per PSU was calculated. In urban areas, a specific street was chosen randomly; in rural areas, the sampling point was selected randomly either from a list of streets (if such a list was available) or from a list of landmarks (church, library, bus stop, etc.). Households were selected using a random route procedure. In urban areas and in rural areas where a list of streets was available, the household with the lowest number in the street was selected as the sampling point. In the other rural areas, the household closest to the chosen landmark was selected as the starting point. After a successful interview, five households were skipped in urban areas and three in rural areas. After unsuccessful interview attempts, the interviewer simply proceeded to the next household without skipping. Within a household, individual respondents were selected using the recent birthday method (the interview was carried out with the adult in the household who had the most recent birthday). Three attempts were made to complete the interview with the selected respondent before proceeding to the next household. Quotas were set as independent response targets for each characteristic: targets were pursued per class within each variable, regardless of achievement of the other quota variables.

The survey was conducted between October 27, 2017 and December 30, 2017. The number of completed interviews was between 700 and 3,200 respondents per country, proportional to the population size. Kantar Public oversampled 200 additional smokers in each country to allow a more detailed analysis of the results for smokers. A total of 17,160 smoking and non-smoking participants completed the survey, 10,298 in face-to-face interviews and 6,862 online. Face-to-face respondents were not compensated for their participation. Online respondents were all members of an online panel company and received Reward Points. The number of points awarded for survey completion is based on survey length, complexity, and incidence rate. Once a points threshold is reached, panelists may redeem their points for online gift certificates or merchandise. Each country has its own unique catalog.

Data analyses were done using SPSS (IBM Corp, Version 24). Descriptive analyses were calculated for all variables. A rim weighting procedure was run against the population figures from the most recent national census to construct weight variables, with the procedure executed separately for each country. Rim weighting consists of iterations: sample counts for each weight variable were adjusted to fit the actual population proportions (marginal percentages) using as the initial values the result of the previous adjustment. The weighting strategy was designed to correct any misbalance following field work in terms of the three original quota targets (age, gender, and region). The statistical z-test was used to find significant differences in proportions among independent samples of smokers and never-smokers.

## Results

The observed prevalence of smoking in adults 18 years and older in this survey ranged from an age-adjusted high of 57.5% in Lebanon to 8.4% in New Zealand among men, and from 48.4% in Lebanon to 1.0% in India among women ( Table 1). The majority of smokers in all countries were between the ages of 25 and 54 ( Table 2).

**Table 1.** Prevalence of smoking by country and sex.

Country	BR	FR	GR	IL	IN	JP	LB	MW	NZ	RU	SA	UK	US
n	1000	1051	1001	502	3127	1000	524	975	1000	1500	1000	1049	1053
% Men	46.8%	48.8%	48.2%	49.2%	49.3%	43.3%	48.4%	44.4%	49.7%	43.2%	44.4%	48.3%	49.4%
% Smokers	9.0%	30.5%	34.6%	16.9%	8.9%	22.3%	52.8%	9.4%	7.3%	26.4%	40.7%	20.9%	18.6%
n	*480*	*496*	*455*	*249*	*1542*	*480*	*253*	*604*	*470*	*635*	*483*	*504*	*519*
% of men who smoke	11.0%	31.5%	37.9%	22.2%	17.1%	25.9%	57.5%	15.6%	8.4%	39.1%	49.0%	26.3%	26.0%
n	*520*	*555*	*546*	*253*	*1585*	*520*	*271*	*371*	*530*	*865*	*517*	*545*	*534*
% of women who smoke	7.2%	29.6%	31.4%	11.9%	1.0%	19.5%	48.4%	4.4%	6.1%	16.7%	34.1%	15.9%	11.3%
n	*168*	*419*	*430*	*150*	*365*	*316*	*338*	*182*	*136*	*514*	*493*	*299*	*255*
% of smokers who are men	57.2%	50.4%	52.9%	64.4%	94.4%	50.4%	52.7%	73.6%	57.6%	64.0%	53.4%	60.6%	69.3%

Data are presented as unweighted n and weighted percentages

BR=Brazil, FR=France, GR=Greece, IL=Israel, IN=India, JP=Japan, LB=Lebanon, MW=Malawi, NZ=New Zealand, RU=Russia, SA=South Africa, UK=United Kingdom, US=United States

**Table 2.** Prevalence of smoking by age and sex.

Country		BR	FR	GR	IL	IN	JP	LB	MW	NZ	RU	SA	UK	US
n		1000	1051	1001	502	3127	1000	524	975	1000	1500	1000	1049	1053
Age 18–24	Men	10.6%	32.1%	48.5%	18.9%	9.3%	34.2%	68.2%	12.1%	4.7%	36.3%	40.8%	51.4%	39.1%
	Women	3.7%	29.4%	19.8%	14.0%	1.1%	26.4%	32.3%	0.7%	3.7%	31.0%	26.2%	25.9%	13.8%
25–39	Men	10.8%	49.0%	44.1%	51.6%	14.8%	33.7%	64.6%	17.3%	7.0%	53.6%	55.7%	48.5%	42.7%
	Women	7.2%	34.7%	40.2%	16.1%	1.1%	29.0%	50.6%	0.9%	6.4%	27.3%	33.7%	23.3%	14.5%
40–54	Men	13.2%	38.7%	44.7%	20.8%	21.7%	35.5%	58.3%	13.8%	8.2%	45.0%	57.2%	27.3%	20.8%
	Women	5.9%	38.5%	41.1%	13.0%	0.8%	24.0%	57.7%	0.8%	4.9%	18.9%	33.8%	13.7%	12.6%
55–64	Men	13.0%	25.3%	46.3%	10.8%	25.4%	16.7%	44.1%	15.1%	8.5%	28.7%	55.9%	8.9%	11.1%
	Women	10.2%	22.9%	35.7%	2.8%	1.5%	14.1%	50.8%	23.3%	9.3%	7.1%	52.8%	8.3%	8.2%
65+	Men	5.4%	15.8%	19.4%	2.7%	17.3%	19.3%	0.0%	22.1%	10.9%	8.8%	9.4%	4.8%	8.6%
	Women	10.7%	18.7%	15.9%	3.9%	0.0%	9.4%	0.0%	32.9%	7.1%	2.4%	32.3%	8.3%	5.2%

Data are presented as unweighted n and weighted percentages

BR=Brazil, FR=France, GR=Greece, IL=Israel, IN=India, JP=Japan, LB=Lebanon, MW=Malawi, NZ=New Zealand, RU=Russia, SA=South Africa, UK=United Kingdom, US=United States

**Figure 1.**
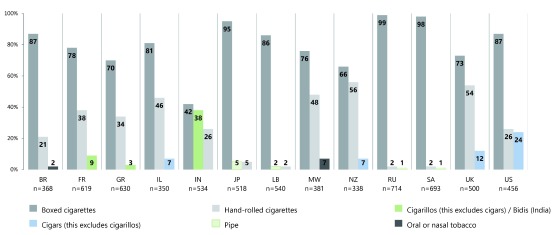
Types of tobacco products used by country. Smokers: Which of the following tobacco products do you use? (multiple answers possible). % top 3 answers. Data are presented as unweighted n and weighted percentages. BR=Brazil, FR=France, GR=Greece, IL=Israel, IN=India, JP=Japan, LB=Lebanon, MW=Malawi, NZ=New Zealand, RU=Russia, SA=South Africa, UK=United Kingdom, US=United States.

**Figure 2.**
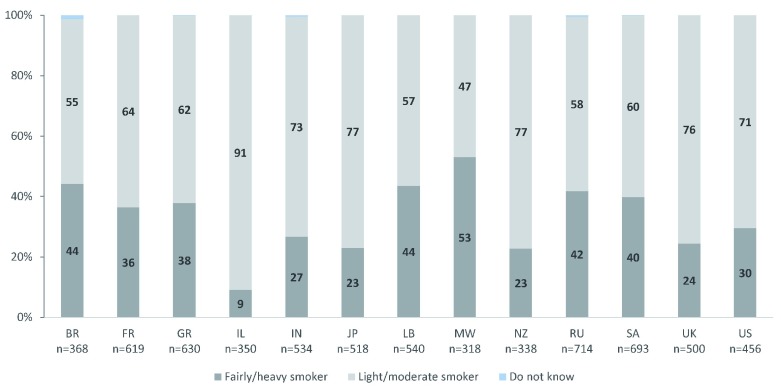
Smokers' self-categorization of amount of smoking by country. Data are presented as smokers unweighted n and weighted percentages. BR=Brazil, FR=France, GR=Greece, IL=Israel, IN=India, JP=Japan, LB=Lebanon, MW=Malawi, NZ=New Zealand, RU=Russia, SA=South Africa, UK=United Kingdom, US=United States. Column percentages may not add up to 100% due to rounding.

**Figure 3.**
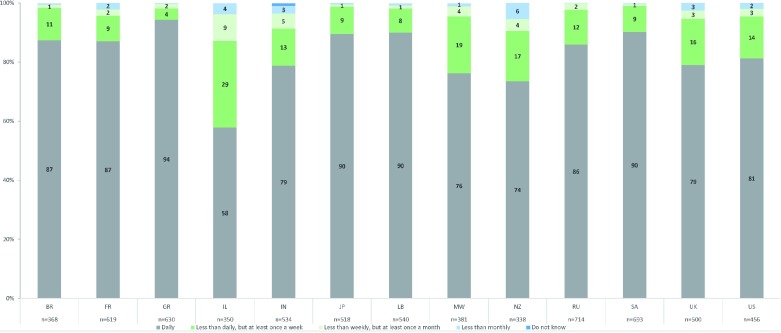
Frequency of smoking by country. Data are presented as smokers unweighted n and weighted percentages. BR=Brazil, FR=France, GR=Greece, IL=Israel, IN=India, JP=Japan, LB=Lebanon, MW=Malawi, NZ=New Zealand, RU=Russia, SA=South Africa, UK=United Kingdom, US=United States. Column percentages may not add up to 100% due to rounding.

Boxed cigarettes were the preferred tobacco product of choice in all countries, followed by hand-rolled cigarettes/roll-your-own (RYO) in most countries ( Figure 1). In almost all countries, the majority of smokers surveyed (47-91%) viewed themselves as light or moderate smokers ( Figure 2). Between 58% (Israel) and 94% (Greece) smoked daily ( Figure 3).

**Figure 4.**
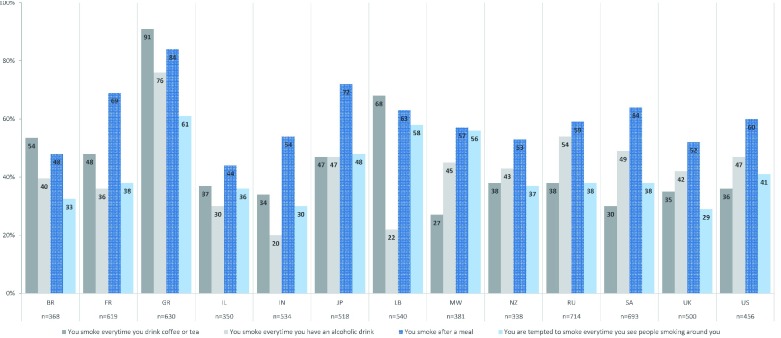
Smoking is associated with daily routines throughout the world. Smokers, multiple answers possible, % yes. Data are presented as unweighted n and weighted percentages. BR=Brazil, FR=France, GR=Greece, IL=Israel, IN=India, JP=Japan, LB=Lebanon, MW=Malawi, NZ=New Zealand, RU=Russia, SA=South Africa, UK=United Kingdom, US=United States.

In aggregated data across all countries, a majority of smokers smoked after meals (62.2%), and many also smoked every time they had coffee or tea (46.1%), or an alcoholic beverage (43.6%). Smokers were also tempted to smoke when they saw others smoking nearby (41.9%). Figure 4 shows the breakdown for these routines by country.

**Figure 5a–d.**
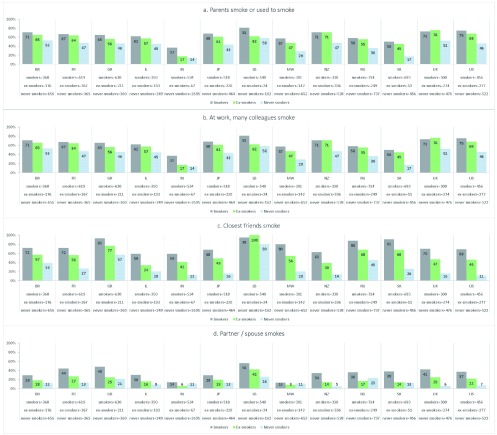
The social environment of smokers, ex-smokers, and never smokers. Data are presented as unweighted n and weighted percentages for % applicable. BR=Brazil, FR=France, GR=Greece, IL=Israel, IN=India, JP=Japan, LB=Lebanon, MW=Malawi, NZ=New Zealand, RU=Russia, SA=South Africa, UK=United Kingdom, US=United States.

In almost all countries, more current smokers than ex-smokers or nonsmokers were surrounded by people who also smoked, including parents, spouse/partner, close friends and colleagues ( Figure 5a–d).

Nearly two-thirds or more of smokers in all countries consider themselves addicted to cigarettes ( Table 3). Smokers were also asked if they smoked a few minutes after waking up (15% Israel to 59% Malawi) and if they could not go two hours without smoking (13% Israel to 37% Lebanon). Additionally, more than 60% of smokers and ex-smokers in India, Malawi and Brazil, had bought cigarettes when they knew the money could be spent better on household essentials like food.

**Table 3.** Perception and markers of addiction to smoking.

Country		BR	FR	GR	IL	IN	JP	LB	MW	NZ	RU	SA	UK	US
Smokers, n		368	619	630	350	534	518	540	381	338	714	693	500	456
Do you consider yourself addicted to cigarettes?	Total "addicted"	85.3%	87.4%	90.0%	68.7%	64.9%	90.9%	71.9%	74.0%	79.9%	85.6%	76.3%	79.0%	79.5%
You smoke a few minutes after waking up	Yes	48.9%	32.9%	41.3%	14.7%	48.3%	49.7%	39.6%	53.4%	38.2%	35.7%	59.3%	35.2%	47.6%
You can't spend 2 hours without smoking	Yes	34.1%	19.8%	38.0%	13.3%	24.5%	18.2%	37.3%	32.6%	19.7%	27.7%	31.4%	21.2%	19.1%
Have you ever spent money on cigarettes that you knew would be better spent on household essentials like food? (yes)	Yes	86.8%	46.9%	44.4%	29.0%	60.2%	20.5%	29.0%	60.5%	36.1%	51.2%	40.6%	33.8%	37.2%

Data are presented as unweighted n and weighted percentages

BR=Brazil, FR=France, GR=Greece, IL=Israel, IN=India, JP=Japan, LB=Lebanon, MW=Malawi, NZ=New Zealand, RU=Russia, SA=South Africa, UK=United Kingdom, US=United States

Table 4 shows smokers’ quitting attempts and intentions to quit. In most countries a majority of smokers said they had tried to quit at least once, with more than half reporting multiple attempts. The range was similar when smokers were asked if they planned to quit, with 78% of smokers in New Zealand responding “yes” compared to 25% in Lebanon. Many of those planning to quit, however, indicated it would be in the future, beyond six months. Of those who were not planning to quit, up to half had previously attempted to quit.

**Table 4.** Smoking cessation attempts and future intentions.

Country	BR	FR	GR	IL	IN	JP	LB	MW	NZ	RU	SA	UK	US
Smokers, n	368	619	630	350	534	518	540	381	338	714	693	500	456
Have tried to quit at least once	71.4%	72.4%	51.3%	68.7%	53.3%	69.7%	32.2%	61.3%	81.4%	67.9%	35.4%	52.6%	51.4%
Planning on quitting	69.6%	72.2%	39.0%	72.0%	51.1%	60.1%	24.9%	71.8%	77.9%	53.7%	40.7%	50.5%	49.4%
Planning on quitting in the future, beyond six months	32.7%	31.5%	31.7%	34.9%	17.9%	48.0%	20.8%	23.6%	35.8%	36.3%	27.1%	30.1%	29.9%
Percent of those not planning to quit who had previously attempted to quit	56.1%	38.3%	38.6%	50.2%	27.3%	41.9%	23.9%	45.2%	57.2%	50.5%	14.9%	34.1%	33.1%

Data are presented as unweighted n and weighted percentages

BR=Brazil, FR=France, GR=Greece, IL=Israel, IN=India, JP=Japan, LB=Lebanon, MW=Malawi, NZ=New Zealand, RU=Russia, SA=South Africa, UK=United Kingdom, US=United States

**Figure 6.**
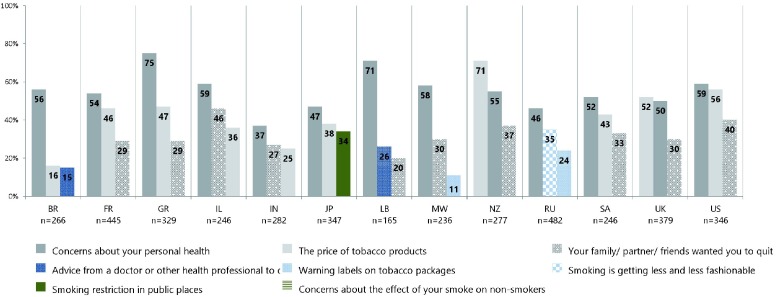
Smokers who have tried to quit: Which of the following factors encouraged you to quit smoking or to consider quitting? % - top three answers. Data are presented as unweighted n and weighted percentages. BR=Brazil, FR=France, GR=Greece, IL=Israel, IN=India, JP=Japan, LB=Lebanon, MW=Malawi, NZ=New Zealand, RU=Russia, SA=South Africa, UK=United Kingdom, US=United States.

**Figure 7.**
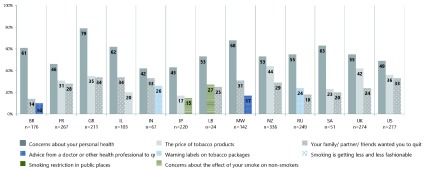
Ex-smokers' leading factors for quitting. Data are presented as unweighted n and weighted percentages. BR=Brazil, FR=France, GR=Greece, IL=Israel, IN=India, JP=Japan, LB=Lebanon, MW=Malawi, NZ=New Zealand, RU=Russia, SA=South Africa, UK=United Kingdom, US=United States.

Survey participants listed concern about personal health the most often as a reason for quitting smoking, except in New Zealand and the UK where more smokers cited the price of tobacco products ( Figure 6 and Figure 7). In addition to price, pressure from family, partner, and/or friends was another leading driver for both smokers and ex-smokers. There were some differences in motivation between smokers and ex-smokers, notably current Russian smokers find that smoking is becoming less fashionable, whereas ex-smokers’ concern for the impact of secondhand smoke made the top three factors for quitting in Japan and Lebanon. The impact of warning labels was among the top three reasons for quitting in India, Malawi, and Russia.

**Figure 8.**
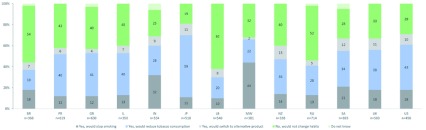
Smokers' responses to a hypothetical price increase on tobacco products. Smokers: would an increase in tobacco price have an effect on your current smoking habit?. Data are presented as unweighted n and weighted percentages. BR=Brazil, FR=France, GR=Greece, IL=Israel, IN=India, JP=Japan, LB=Lebanon, MW=Malawi, NZ=New Zealand, RU=Russia, SA=South Africa, UK=United Kingdom, US=United States. Column percentages may not add up to 100% due to rounding.

When further questioned about the impact of price, a majority of smokers in 10 of the 13 countries surveyed said they would stop smoking, reduce their tobacco consumption, or switch to alternative products if the price of tobacco increased ( Figure 8).

**Figure 9.**
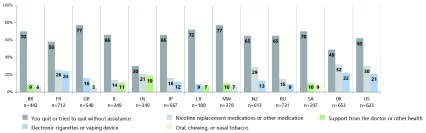
Methods used to try to quit smoking. Ex-smokers, or smokers who have tried to quit. Data are presented as unweighted n and weighted percentages. FR=France, UK=United Kingdom, US=United States.

Figure 9 highlights the main methods used by smokers and ex-smokers to quit or try to quit. The majority used no type of assistance. A minority of participants in all countries reported receiving support from a healthcare professional, other specialist, or from a specialized stop-smoking clinic when trying to quit.

**Figure 10.**
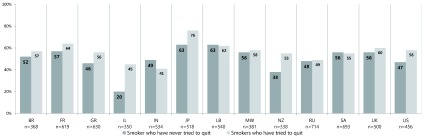
Smokers perceived need for assistance to quit smoking. Smokers: Let's imagine that you have to give up smoking completely tomorrow. Which of the following statements would best apply to you? (% - would need to seek assistance). Data are presented as unweighted n and weighted percentages. BR=Brazil, FR=France, GR=Greece, IL=Israel, IN=India, JP=Japan, LB=Lebanon, MW=Malawi, NZ=New Zealand, RU=Russia, SA=South Africa, UK=United Kingdom, US=United States.

**Figure 11.**
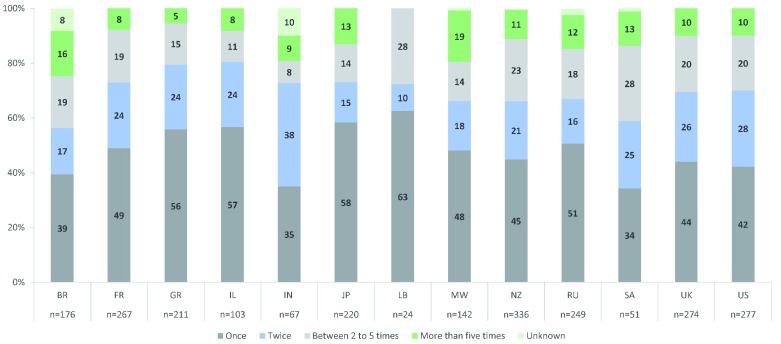
Number of attempts prior to quitting smoking successfully. Ex-smokers: How many times did you try to quit smoking before you were successful? Data are presented as unweighted n and weighted percentages. BR=Brazil, FR=France, GR=Greece, IL=Israel, IN=India, JP=Japan, LB=Lebanon, MW=Malawi, NZ=New Zealand, RU=Russia, SA=South Africa, UK=United Kingdom, US=United States. Column percentages may not add up to 100% due to rounding.

In most countries, more than half of the smokers who had previously tried to quit and failed reported they would need assistance to quit ( Figure 10). Among ex-smokers, up to 40% needed three or more attempts to quit successfully ( Figure 11).

Smokers in almost all countries surveyed were less likely to describe their health as “excellent” or “very good” compared to never smokers ( Table 5). More smokers in every country reported that they drank “too much” alcohol, and in most countries they reported feeling stressed in their personal or work lives more than never smokers ( Table 5). Results on other variables such as weight, physical activity, healthy food consumption, and environmental factors were mixed between smokers and never smokers across countries (not shown).

**Table 5.** Smokers compared with non-smokers, view of health, health behaviors, and environment.

Country		Brazil			France			Greece			Israel			India			Japan			Lebanon			Malawi			New Zealand			Russia			South Africa			United Kingdom			United States		
		Smokers	Never Smokers	Z- value	Smokers	Never Smokers	Z- value	Smokers	Never Smokers	Z- value	Smokers	Never Smokers	Z- value	Smokers	Never Smokers	Z- value	Smokers	Never Smokers	Z- value	Smokers	Never Smokers	Z- value	Smokers	Never Smokers	Z- value	Smokers	Never Smokers	Z- value	Smokers	Never Smokers	Z- value	Smokers	Never Smokers	Z- value	Smokers	Never Smokers	Z- value	Smokers	Never Smokers	Z- value
n		368	656		619	365		630	360		350	249		534	2695		518	464		540	162		381	652		338	528		714	737		693	456		500	476		456	522	
In general, how would you describe your health?	Very good / Excellent	13.6%	29.4%	-4.214	16.0%	27.5%	-3.884	50.6%	59.5%	-2.505	36.1%	53.0%	-3.265	18.9%	26.8%	-3.278	8.0%	11.5%	-1.579	33.2%	61.1%	-5.880	34.1%	50.0%	-3.607	19.1%	44.1%	-5.357	11.5%	11.8%	-0.192	45.2%	62.8%	-5.381	18.8%	33.7%	-4.490	32.0%	50.2%	-4.907
You tend to drink alcohol a bit too much	Total "Agree"	29.1%	15.0%	4.265	27.0%	10.1%	5.989	18.3%	4.6%	5.937	13.8%	4.8%	3.181	22.0%	6.6%	9.678	42.1%	13.1%	9.154	8.9%	4.4%	1.753	38.4%	6.8%	10.330	27.5%	12.9%	4.168	12.3%	3.6%	5.861	33.4%	13.1%	7.094	34.7%	14.1%	6.736	28.7%	9.6%	7.000
You often feel stressed at work	Total "Agree"	58.9%	48.7%	2.287	46.4%	40.4%	1.671	65.5%	48.8%	4.297	64.2%	52.7%	2.240	36.3%	30.6%	2.132	52.9%	41.8%	3.005	71.4%	65.6%	1.286	44.6%	32.4%	2.829	42.8%	39.8%	0.639	43.4%	29.7%	4.761	36.7%	19.8%	5.347	43.5%	31.1%	3.477	41.3%	34.1%	1.987
You currently suffer from stress in your personal life	Total "Agree"	60.8%	51.2%	2.224	48.5%	43.0%	1.551	60.4%	49.2%	3.174	56.5%	48.5%	1.552	41.1%	35.3%	2.202	54.3%	47.2%	1.945	75.3%	63.6%	2.706	53.2%	41.1%	2.723	53.8%	40.9%	2.726	27.7%	24.8%	1.135	34.5%	23.6%	3.606	51.9%	43.8%	2.194	56.9%	49.7%	1.922

Data are presented as unweighted n and weighted percentages

Results are based on two-sided tests with significance level 0.05, Z ≥ 1.96 or Z ≤ −1.96

BR=Brazil, FR=France, GR=Greece, IL=Israel, IN=India, JP=Japan, LB=Lebanon, MW=Malawi, NZ=New Zealand, RU=Russia, SA=South Africa, UK=United Kingdom, US=United States

Participants stated they were well informed (very well informed or rather well informed) about the impact of smoking on their health (67% Malawi to 96% US) ( Table 6). Further, they agreed (totally agree or tend to agree) that smoking was harmful to their own health (69% India to 96% Brazil) and to the health of others (66% India to 95% Greece). A majority of smokers were able to identify multiple conditions associated with smoking such as lung cancer and heart disease (not shown).

**Table 6.** Perception of impact of smoking on health.

Country		BR	FR	GR	IL	IN	JP	LB	MW	NZ	RU	SA	UK	US
Smokers, n		368	619	630	350	534	518	540	381	338	714	693	500	456
To what extent do you feel well informed or not about smoking and its impact on your health?	Well informed	82.9%	92.3%	94.2%	95.3%	68.2%	86.0%	94.5%	67.4%	95.7%	91.3%	86.4%	93.1%	96.0%
Your smoking is harmful for your health	Agree	96.2%	88.8%	95.3%	89.2%	69.3%	85.5%	91.5%	89.6%	86.7%	88.9%	81.3%	83.5%	86.1%
In some cases, your smoking could harm others around you	Agree	94.0%	79.3%	94.6%	88.8%	65.7%	82.2%	91.8%	78.9%	71.8%	85.1%	82.1%	72.5%	75.9%

Data are presented as unweighted n and weighted percentages

BR=Brazil, FR=France, GR=Greece, IL=Israel, IN=India, JP=Japan, LB=Lebanon, MW=Malawi, NZ=New Zealand, RU=Russia, SA=South Africa, UK=United Kingdom, US=United States

**Figure 12.**
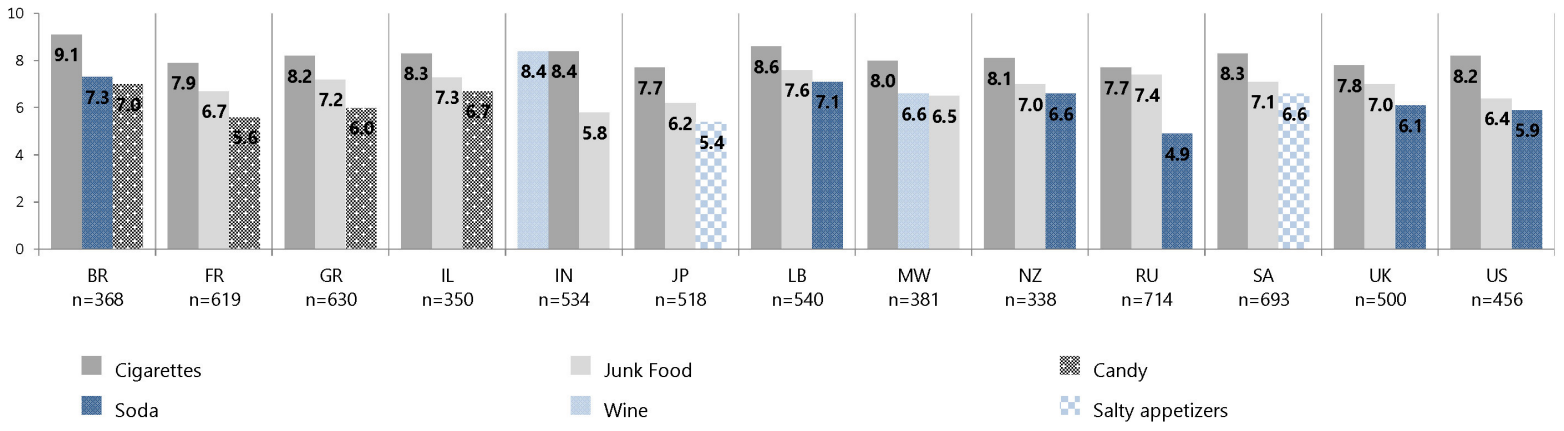
Smokers' rating of the harmfulness of various products. Smokers: On a scale from 1 to 10, where 1 means not harmful to your health and 10 means very harmful to your health, to what extent do you think a moderate daily use of the following products can harm your health? (Ten point scale average: top three answers per country). Data are presented as unweighted n and weighted percentages. BR=Brazil, FR=France, GR=Greece, IL=Israel, IN=India, JP=Japan, LB=Lebanon, MW=Malawi, NZ=New Zealand, RU=Russia, SA=South Africa, UK=United Kingdom, US=United States.

**Figure 13.**
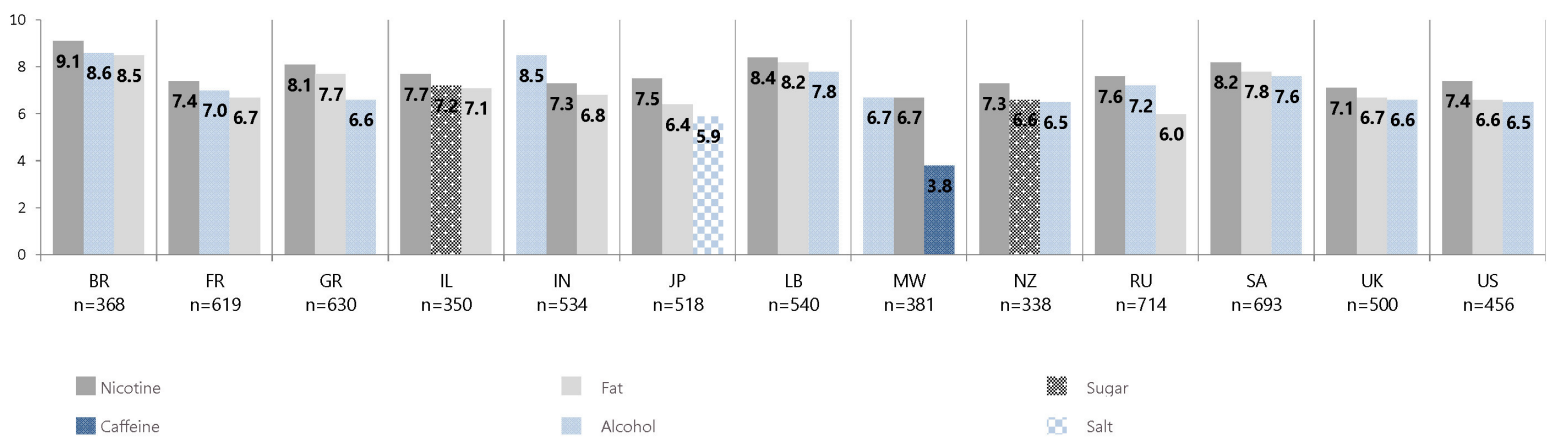
Smokers' rating of the harmfulness of various substances. Smokers: On a scale from 1 to 10, where 1 means not harmful to your health and 10 means very harmful to your health, to what extent do you think a moderate daily use of the following substances can harm your health? (Ten point scale average: top three answers per country). Data are presented as unweighted n and weighted percentages. BR=Brazil, FR=France, GR=Greece, IL=Israel, IN=India, JP=Japan, LB=Lebanon, MW=Malawi, NZ=New Zealand, RU=Russia, SA=South Africa, UK=United Kingdom, US=United States.

When asked to rate the harmfulness of cigarettes and other products such as wine, soda drinks, candy, junk food, and salty appetizers on one’s health, smokers as a group in all countries gave cigarettes the highest average harm rating compared to other products ( Figure 12). When asked to rate the harmfulness of moderate daily use of the following substances: alcohol, caffeine, fat, nicotine, salt, and sugar, nicotine was given the highest average harm rating ( Figure 13).

**Figure 14.**
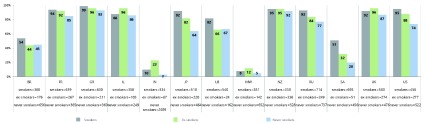
Awareness of ENDS. Have you heard of the following products? Electronic cigarettes, e-cigarettes or vaping devices. % - yes. Data are presented as unweighted n and weighted percentages. BR=Brazil, FR=France, GR=Greece, IL=Israel, IN=India, JP=Japan, LB=Lebanon, MW=Malawi, NZ=New Zealand, RU=Russia, SA=South Africa, UK=United Kingdom, US=United States. ENDS=Electronic Nicotine Delivery Systems.

Most participants in high income countries had heard of electronic cigarettes, e-cigarettes or vaping devices (ENDS, including devices with or without nicotine) ( Figure 14). Awareness of heat-not-burn/heated tobacco products (HTP) among smokers was relatively low in all countries aside from Japan (86%) and Israel (52%).

**Figure 15.**
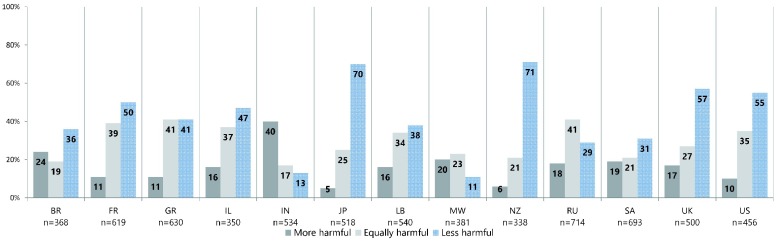
Perception of risk associated with ENDS versus combustible cigarettes. Smokers: Do you think smoking e-cigarettes and vaping devices is more or less harmful than smoking regular cigarettes? Data are presented as unweighted n and weighted percentages. BR=Brazil, FR=France, GR=Greece, IL=Israel, IN=India, JP=Japan, LB=Lebanon, MW=Malawi, NZ=New Zealand, RU=Russia, SA=South Africa, UK=United Kingdom, US=United States. ENDS=Electronic Nicotine Delivery Systems.

The perception by smokers regarding the relative harmfulness of ENDS compared with regular cigarettes was mixed, with many choosing not to answer, and the majority of smokers in only four countries believing that ENDS were less harmful than regular cigarettes ( Figure 15). Data are also available regarding perceptions of relative harm to others through second hand smoke and vapor; of relative addictiveness to ENDS; and of association of nicotine in ENDS with various health conditions.

**Figure 16.**
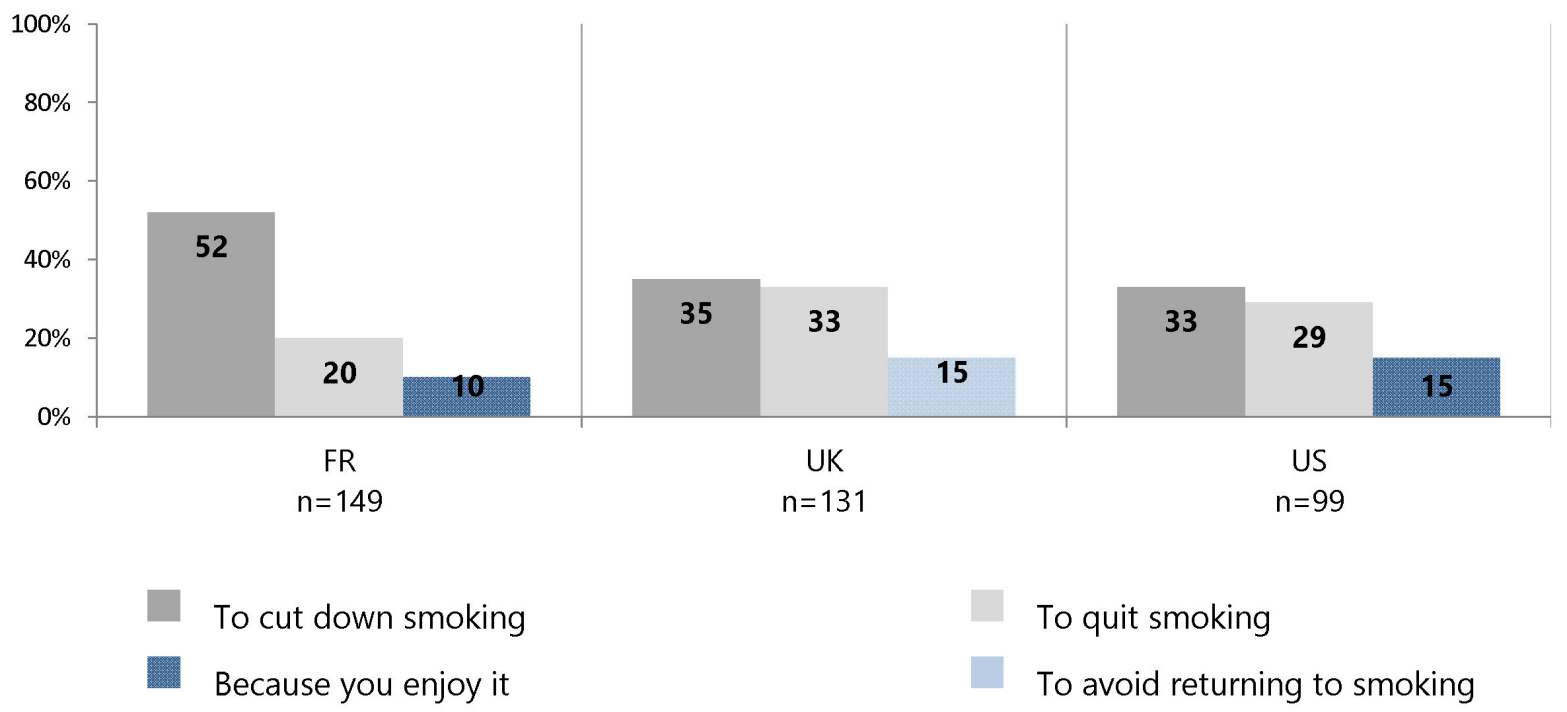
Reasons for regular ENDS use. Regular users of ENDS. % - top 3 answers per country. Data are presented as unweighted n and weighted percentages. FR=France, UK=United Kingdom, US=United States. ENDS=Electronic Nicotine Delivery Systems.

**Figure 17.**
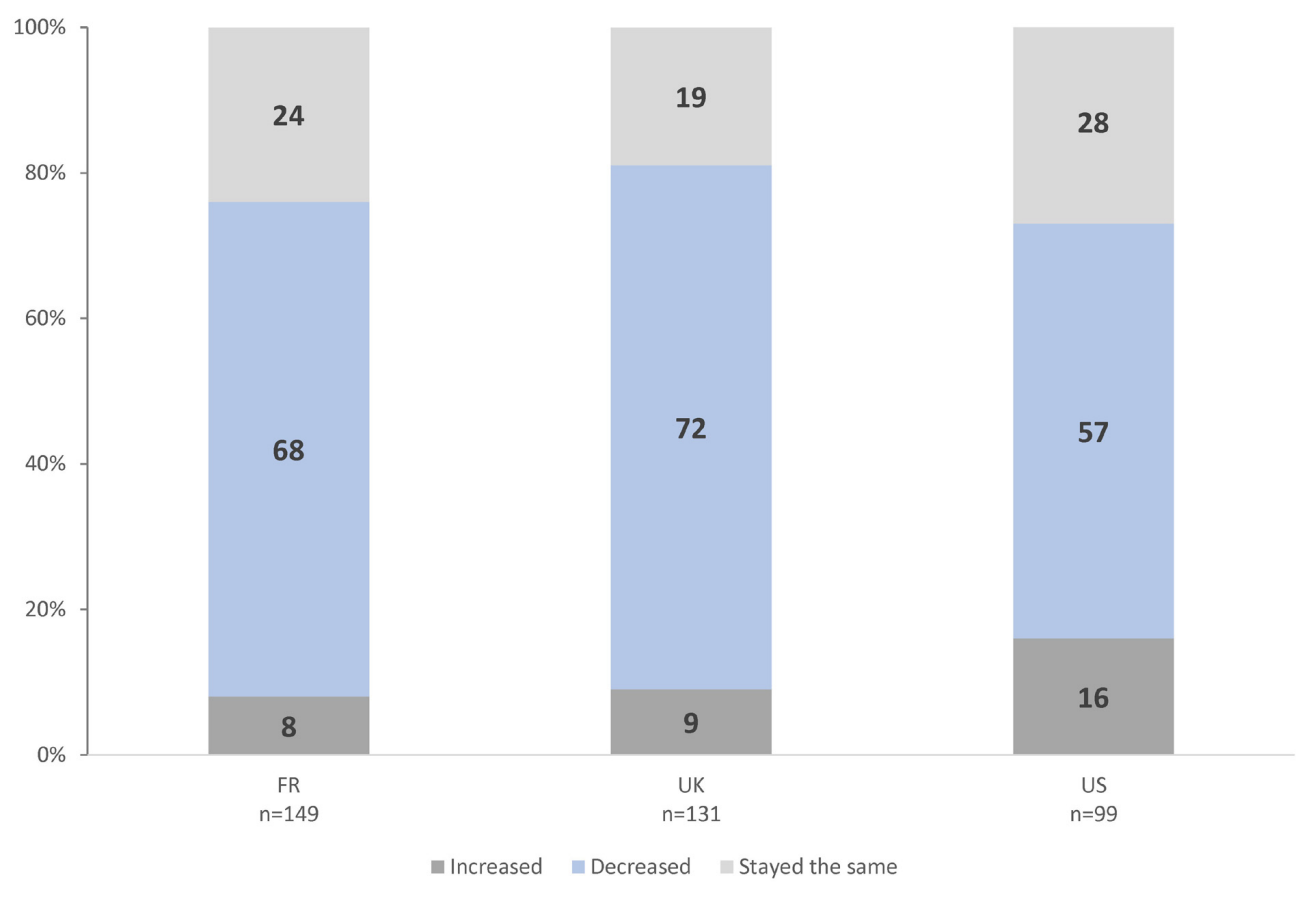
Impact of regular ENDS use on tobacco consumption. Since you started using these products, would say your tobacco consumption has increased, decreased or stayed the same? Data are presented as unweighted n and weighted percentages. Column percentages may not add up to 100% due to rounding. FR=France, UK=United Kingdom, US=United States. ENDS=electronic nicotine delivery systems.

Analysis of ENDS users was limited to France, UK, and US since the number of self-identified regular users of ENDS was too low in the remaining countries. The most often cited reasons to use ENDS were for decreasing or quitting smoking, however, among the top 3 reasons in the US and France was also the use of ENDS for enjoyment ( Figure 16). More than half of these participants reported their tobacco consumption had decreased since regular use of ENDS ( Figure 17). Additionally, one-fifth of these regular users indicated they choose products that do not contain nicotine (France 23.4%, UK 22%, US 20%).

## Discussion

The FSFW global survey of smoking behavior and perceptions of more than 17,000 people in 13 countries identifies issues that will guide future efforts to stop smoking worldwide.

The results are consistent with other findings that show more male smokers, with women catching up in some countries and age groups ^4^. We report a predominance of boxed cigarette use, while RYO is the second most common tobacco product in almost all countries, used by 2%–56% of smokers. The lower price of RYO compared to boxed cigarettes is cited by smokers and supported by price analyses as a reason for RYO use ^11, 12^. Our results show the highest number of RYO users being in NZ and UK, the two countries with the most expensive boxed cigarettes in this survey and which also had the most participants citing cost as a consideration for quitting smoking ^13^.

Taxing tobacco products is considered a best practice under the World Health Organization (WHO) Framework Convention on Tobacco Control (FCTC) ^14^ with global data demonstrating that increasing taxes on tobacco products is the most effective approach to reducing their use and encouraging users to quit ^1^. A majority of smokers in this survey indicated they would change their smoking habits (stop smoking, reduce their tobacco consumption, or switch to alternative products) if the price of tobacco increased.

There is a concern that policies intended to incentivize smokers to quit are instead moving smokers away from highly taxed boxed cigarettes to a potentially more harmful alternative. RYO cigarettes vary in composition but have been shown to cause comparable exposure to known and suspected carcinogens ^15^. Nevertheless, there is an erroneous belief among many users that RYO are a healthier alternative to boxed cigarettes ^11^. An association of RYO users with lower educational or socioeconomic status, as well as a history of less stringent warning labels, may contribute to RYO users being less well informed of RYO tobacco risks ^11^. More work is needed to address the continued rise of RYO use across countries.

A majority of smokers in this survey generally characterize themselves as light or moderate smokers and most smoked daily. Nearly two-thirds or more of smokers considered themselves addicted to cigarettes. Across all countries, smokers associated smoking with daily routines. Smokers are surrounded by other smokers, and their smoking is tied to socially relevant activities such as meals, or drinking, whether it be coffee, tea, or alcohol.

The survey results indicate in most countries two-thirds of smokers try to quit without assistance, similar to rates reported elsewhere ^16^. Behavioral therapies (group ^17^ and individual ^18^) and social supports ^19^ that would address the deeply ingrained daily routines and social interactions of smokers have been shown to increase quit rates, however in a comparison of 22 national guidelines on smoking cessation, the recommended content and delivery of these therapies varies widely ^20^. Although many smokers eventually quit without assistance ^21^, on any given quit attempt, the success without assistance for remaining abstinent for at least 6–12 months is about 3–5% ^22^.

Using the assistance of a healthcare professional or specialized stop-smoking clinic or specialist increases the likelihood of short and long-term quitting primarily through the advice to use, or prescription of, cessation medications ^21, 23^. Nicotine replacement therapies (NRT) increase the rate of quitting by 50–60% ^24^, however, given the low rates of abstinence alone, this translates to an absolute efficacy increase in most populations of about 3%. A national sample of US adult smokers found just 40% of current smokers had ever used NRT such as patches, gum, or other products approved for smoking cessation, even though nearly all knew about these products ^25^. In this survey, fewer than 20% sought specialized assistance and only a third or fewer smokers had tried NRT or other medications when trying to quit.

Of those that failed in previous quit attempts, 14% (South Africa) to 57% (New Zealand) indicated they are not interested in trying again, underscoring the need for better and more comprehensive smoking cessation information and programs to increase quit success rates at the outset. Understanding the profiles of successful ex-smokers and of current smokers interested in quitting, the prime motivators to quit, and who is amenable to assistance can improve policies and outreach efforts for smokers seeking to quit.

Smokers are largely aware of the health consequences of smoking and it is the most often cited reason for quitting among ex-smokers, and for current smokers in most countries.

However, while smokers are broadly correct in acknowledging the harm of cigarettes, many are confused as to the source of the harm. When asked to rate the harmfulness of cigarettes, most rate cigarettes as more harmful than other products such as wine, junk food, soda, salty appetizers, and candy. However, when asked to rate the harmfulness of moderate daily use of nicotine to their own health, smokers again rate nicotine very high, exceeding or matching every other substance (salt, fat, sugar, alcohol, caffeine). In a 2015 national US survey, nearly one-half believed nicotine in cigarettes is the main cause of smoking related cancer, and another 24% were unsure ^26^. Other surveys report similar misconceptions about nicotine in NRT, with 21% of smokers believing the patch is associated with heart problems ^25^, and two thirds of a pool of smokers and ex-smokers agreeing or unsure that “stop-smoking products with nicotine are just as harmful as cigarettes” ^27^. Further research to parse out participants’ intentions in rating harmfulness of nicotine is needed, as well as asking about tobacco as a substance for comparison. Misperceptions of the role of nicotine could be limiting public health efforts to curtail smoking, including contributing to low uptake of NRT ^28^ or confusion regarding reduced-risk products ^26^.

Tobacco control includes substitution of higher harm products with lower harm products ^14^. While the consensus holds ENDS are substantially less harmful than traditional cigarettes ^29, 30^, public health messaging regarding use varies. In the UK, ENDS are promoted for smoking cessation whereas the WHO recommends regulatory measures to protect against possible health risks ^28^. Some countries, including several in this study, have restricted or banned sale and/or possession of ENDS ^31^. These competing messages regarding ENDS appear to add to the misunderstanding of the role of nicotine ^32^. In two-thirds of surveyed countries there was a very high level of awareness of ENDS, however, many smokers were unable or unwilling to categorize whether ENDS were more, less, or equally harmful to health compared with regular cigarettes.

ENDS users in this survey most frequently cite adopting ENDS to quit or cut down on smoking. In a longitudinal survey of US adult smokers, substituting ENDS for some cigarettes when trying to quit was a method used more often than the nicotine patch or gum, or other smoking cessation medications approved by the US Food and Drug Administration, with one-quarter of the most recent quit attempts replacing all cigarettes with ENDS ^33^. Compared to other nicotine-non-tobacco products ENDS most closely simulate smoking regular cigarettes in how they are used. The variety of products allow users to customize their experience in terms of flavor and amount of nicotine which could further enhance ENDS as a replacement for traditional cigarettes.

A majority of ENDS users in countries with sufficient sample size in this survey report decreased tobacco consumption since starting ENDS. Studies to date tend to focus on ENDS use and quit rates as absolutes rather than assessing the relative harm reduction, especially when considering the risk status of a smoker versus a never smoker. Models that consider a public health perspective of ENDS are positive, with 1.6 million or more fewer premature deaths over 10 years, even in scenarios where not all smokers quit when using ENDS, some never smokers become ENDS users, and more harm is attributed to ENDS than has been currently found ^34^. Further investigation and research of ENDS use and other alternatives along this spectrum of harm reduction is needed.

This survey is limited by potential response bias in those choosing to participate, and reporting bias as there were no external or objective validations. Although the survey results were weighted according to population figures, the sampling was not strictly designed to estimate overall smoking prevalence. Additionally, while the countries included in the survey were chosen to represent a range of income levels and smoking prevalence, generalizability to other countries may be limited by cultural norms and regulations. There may have been differences between surveys administered face-to-face versus online. Despite attention to using previous surveys as a guide to create and pilot the survey, several deficiencies emerged such as quantifying the number of cigarettes smoked. Future surveys will seek to include more questions on the understanding of the role of nicotine, as well as comparisons to other substances, including tobacco. Questions regarding the use of ENDS will eliminate the word “smoking” to reduce confusion regarding the use of tobacco versus non-tobacco products and include more detailed categories for frequency of use.

## Conclusion

This global survey highlights several areas of global smoking behavior and perceptions that need particular attention, namely the deeply social and behavioral aspects of smoking, the inadequacy of current efforts to promote quitting, the role of RYO cigarettes, and the confusion many smokers have regarding tobacco and nicotine products, including ENDS.

This survey report represents the initial piece of FSFW’s work to improve global health and end smoking in this generation.

FSFW is committed to funding research, promoting innovation, and supporting collaborative initiatives to accelerate progress in reducing harm and death from smoking worldwide. To this end, data from this survey are available online for further analyses, and FSFW welcomes input for follow-up surveys.

## Data availability

### Underlying data

Open Science Framework: Global Poll 2018, https://doi.org/10.17605/OSF.IO/X4GQZ ^35^

Data are available under the terms of the Creative Commons Zero “No rights reserved” data waiver (CC0 1.0 Public domain dedication).

### Extended data

Kantar Public methodology report and 81-question quantitative survey: https://amadashboards.com/kp/eu_smokefree/Doc/FSFW%20-%20State%20of%20Smoking%20Survey%20-%20Method%20statement.pdf
