# Supplementary material for: Tobacco smoking and nicotine delivery alternatives: patterns of product use and perceptions in 13 countries
Source: F1000Res. 2019 May 13;8:80. Originally published 2019 Jan 21. [Version 2] doi: 10.12688/f1000research.17635.2 (PMC6518446; doi:10.12688/f1000research.17635.2)
Supplement: Peer review history

# Peer review: Reviewer response for version 2

**DOI:** 10.5256/f1000research.21071.r48393

**Published:** 2019-05-14

**Authors:** K. Michael Cummings

**Affiliations:** 1 Department of Psychiatry & Behavioral Sciences, Hollings Cancer Center, Medical University of South Carolina, Charleston, USA

The reviewers have done a commendable job responding to my previous feedback on their paper. I have no further comments to offer and would recommend that the paper be indexed.

I have read this submission. I believe that I have an appropriate level of expertise to confirm that it is of an acceptable scientific standard.

# Peer review: Reviewer response for version 1

**DOI:** 10.5256/f1000research.19285.r46336

**Published:** 2019-04-01

**Authors:** Axel Klein

**Affiliations:** 1 Global Drug Policy Observatory, University of Swansea, Swansea, UK

This is a very well designed and exhaustive study of attitudes among smoking/vaping populations and explores some of the obstacles to changing behaviour among different cohorts of smokers in different countries. There are interesting variations between for instance Lebanon where high levels of smoking continue to suggest a 'normalised' view of smoking particularly among adult males and countries where smoking is waning and, as the Russian informants reports, no longer fashionable. 

 This formulation is important as it suggests that there are population groups that are not influenced by any of the established means of suasion - information provision on negative health effect, appeal and reconstruction of self image. It undermines both the conceptual model of homo oeconomicus, the rational, means/ends calculating rational analyst or the affective, fashion oriented, decision maker. 

 Interesting data is also coming up about the attitudes to ENDS as an alternative delivery vehicle for tobacco. First, there is a deep rooted misunderstanding of adverse health effects of smoking. Large sections of the population (including physicians) continue to believe that it is the nicotine content of tobacco that is carcinogenic. It is this misconception that may set a block to transitioning to vaping mechanisms as people may think that vaping is just as dangerous because nicotine is not excluded.

 There emerges then a strong policy implication. Public health agencies must become more assertive in pressing the case for vaping. Moreover, the campaign by some lobbies with a view to stigmatize all nicotine use behaviour is not only discriminatory, but also highly irresponsible in public health terms by raising resistance to behaviour change. 

 Through the sheer scale of the research large areas in the smoking cessation/transition debate are well illustrated. In many ways this is a starting point for correcting policy and practice measures and for driving new qualitative research to get a better understanding of the human beings behind the labels of smoker/vaper/nicotine users/ex-smoker and so on. It is also a good platform from where to explore and construe the narratives of transition, redefinitions, new-nicotine sociality and the search for a renewed definition of pleasure.

I have read this submission. I believe that I have an appropriate level of expertise to confirm that it is of an acceptable scientific standard.

# Peer review: Reviewer response for version 1

**DOI:** 10.5256/f1000research.19285.r44210

**Published:** 2019-02-26

**Authors:** K. Michael Cummings

**Affiliations:** 1 Department of Psychiatry & Behavioral Sciences, Hollings Cancer Center, Medical University of South Carolina, Charleston, USA

This is a nicely written paper with lots of great data. However, the paper can be improved upon by providing information on statistical weighting. The description of how weighting of data from what appears to be convenience samples done in different countries is inadequate. In order to generate samples that are weighted so as to reflect the country specific estimates that are provided requires having some country specific source data for each of the 13 countries for which there are survey estimates presented. The question is what source data were used for each country and how closely do the characteristics of the samples from each country match the gold standard source dataset used to generate sampling weights? The authors may have a technical report that investigates this issue and can provide the evidence needed so that readers can feel confident in the country specific estimates provided. However, the paper seems to lack this information, which is critical.

 I was concerned that the investigators did not seek human subjects review for their surveys. I agree the surveys are low risk. However, I think this is something that the investigators ought to address in subsequent survey work that they might undertake. Many journals will not accept such findings without such certification. It was unclear from the methods write up if subjects were compensated for their time taking the survey which is normally done. Was that the case in the survey described and how did compensation differ between countries?

 Country names should either be spelled out or abbreviations, if used described at least one time in the text (i.e., FR=France, BR=Brazil, etc. - see table 1).

 The paper should include definitions of products as it is likely that in Indian bidis are being referred to as cigarettes.  

 Also, India is a big country, what regions of the country were surveyed, and how does region impact product use (i.e., higher in some places for smokeless vs bidis). 

 How is perceived addiction correlated with regular daily use?

 In Table 3 it appears that those from poor countries are more likely to report using tobacco instead of food. That should be stated.

 In Table 4, add the questions on have tried to quit once with have tried to quit more than once to create an overall percentage who have reported having tried to quit.

 On page 10, the statement that RYO tobacco yields higher tar levels compared to boxed tobacco is untrue and misleading. RYO tobacco is most often commercial tobacco packaged for rolling. RYO tobacco packaged in this way typically has more additives (usually for moisture). Tar and nicotine content is mostly determined by the amount rolled which is highly variable. The one reference cited is old, out of date, and perhaps correct for New Zealand at the time, but not generally true world wide. The industry loves to say RYO tobacco is worse than boxed tobacco and that is simply false.

 Also on page 10, RYO is widely used form of tobacco in many countries - this should be reported. 

 The comment on page 12 assumes health professional advice would be expected to be the same across countries. That is simply not realistic. Thus such comparisons are misleading (See paper by Borland et al, One size does not fit all when it comes to smoking cessation: observations from the International Tobacco Control (ITC) Policy Evaluation Project) ^1^. 

 Table 5 is unreadable.

 On page 17, modelling of ENDS effects on health are only really focused on the US and other high income countries. It is unlikely that ENDS would have much impact in countries with very low smoking rates. Also the term ENDS is out of date and ought to be replaced with a broader term - nicotine vaping products (NVPs).

 The data from the study ought to be made publicly available.

 While lots of interesting descriptive data are provide, the paper loses its value since the authors have not focused the presentation on any themes. I would advise reformatting the paper, and use a fewer number of tables to communicate a few key themes. Additional materials can be placed in supplemental tables/figures. I would also encourage the authors to use figures where possible since this would be easier for readers to follow.

I have read this submission. I believe that I have an appropriate level of expertise to confirm that it is of an acceptable scientific standard, however I have significant reservations, as outlined above.

## References

[1] BorlandRHylandACummingsKMFongGT: One size does not fit all when it comes to smoking cessation: observations from the International Tobacco Control Policy Evaluation Project. *Nicotine Tob Res*.2010;12 Suppl: 10.1093/ntr/ntq140 S1-3 10.1093/ntr/ntq140 20889475PMC2948142

# Response

**Published:** 2019-05-02

**Authors:** Sarah Rajkumar

**Affiliations:** Foundation for a smoke-free world, USA

This is a nicely written paper with lots of great data. However, the paper can be improved upon by providing information on statistical weighting. The description of how weighting of data from what appears to be convenience samples done in different countries is inadequate. In order to generate samples that are weighted so as to reflect the country specific estimates that are provided requires having some country specific source data for each of the 13 countries for which there are survey estimates presented. The question is what source data were used for each country and how closely do the characteristics of the samples from each country match the gold standard source dataset used to generate sampling weights? The authors may have a technical report that investigates this issue and can provide the evidence needed so that readers can feel confident in the country specific estimates provided. However, the paper seems to lack this information, which is critical.

***Response**: The methods statement that is available on our website provides more detailed tables on the weighting estimates, starting on page 16. We provide a link to it at the end of the manuscript. The population distribution was taken from the most recent national census data available. We have added this to the paper as well when describing the weighting procedure.*

I was concerned that the investigators did not seek human subjects review for their surveys. I agree the surveys are low risk. However, I think this is something that the investigators ought to address in subsequent survey work that they might undertake. Many journals will not accept such findings without such certification. It was unclear from the methods write up if subjects were compensated for their time taking the survey which is normally done. Was that the case in the survey described and how did compensation differ between countries?

***Response**: We thank the reviewer for his recommendation to seek IRB approval in our next survey as indeed omitting this could lead to an article being rejected right away.*

*Face-to-face respondents were not compensated for their participation. Online respondents were all members of an online panel company. As such, according to the industry standard, they received Reward Points, to encourage panelists to participate in surveys. Upon completion of a survey, points are deposited into a panelist’s account, which gives instant gratification for survey completion. The number of points awarded for survey completion is based on survey length, complexity, and incidence rate. Once a points threshold is reached, panelists may redeem their points for online gift certificates or merchandise. Each country has its own unique catalogue. We have added this information to the methods section on page 4.*

Country names should either be spelled out or abbreviations, if used described at least one time in the text (i.e., FR=France, BR=Brazil, etc. - see table 1).

***Response**: We have ensured that all abbreviations are spelled out at first use.*

The paper should include definitions of products as it is likely that in Indian bidis are being referred to as cigarettes. 

***Response**: We have added bidis to the description of products that smokers use as indeed they were included for the Indian population.*

Also, India is a big country, what regions of the country were surveyed, and how does region impact product use (i.e., higher in some places for smokeless vs bidis).

***Response**: India was divided into four geographical regions - i.e. North, South, East and West and results were weighted according to the population distribution. One state was randomly sampled from each geographic region. The selected states were: Uttar Pradesh (North), Telangana (South), West Bengal (East) and Gujarat (West). We agree that India is too large and diverse a country to integrate all numbers into one result but our sample size was too small to report meaningful data on a sub-national level. We are amending this in our next poll which will be carried out in 2019 with a much larger sample size in India.*

How is perceived addiction correlated with regular daily use?

***Response**: We have decided to only report descriptive numbers and no correlation numbers or further statistical analyses. While exploring correlations between some of the outcomes would certainly be interesting, adding correlations here would go beyond the scope of the present paper.*

In Table 3 it appears that those from poor countries are more likely to report using tobacco instead of food. That should be stated.

***Response**: We have added this statement to the paragraph where we describe results from Table 3.*

In Table 4, add the questions on have tried to quit once with have tried to quit more than once to create an overall percentage who have reported having tried to quit.

***Response**: Done.*

On page 10, the statement that RYO tobacco yields higher tar levels compared to boxed tobacco is untrue and misleading. RYO tobacco is most often commercial tobacco packaged for rolling. RYO tobacco packaged in this way typically has more additives (usually for moisture). Tar and nicotine content is mostly determined by the amount rolled which is highly variable. The one reference cited is old, out of date, and perhaps correct for New Zealand at the time, but not generally true world wide. The industry loves to say RYO tobacco is worse than boxed tobacco and that is simply false.

***Response**: We have deleted this statement*.

Also on page 10, RYO is widely used form of tobacco in many countries - this should be reported.

***Response**: We report this at the beginning of the results section and the discussion.*

The comment on page 12 assumes health professional advice would be expected to be the same across countries. That is simply not realistic. Thus such comparisons are misleading (See paper by Borland et al, One size does not fit all when it comes to smoking cessation: observations from the International Tobacco Control (ITC) Policy Evaluation Project)1.

***Response**: We mention on page 12 that “in a comparison of 22 national guidelines on smoking cessation, the recommended content and delivery of these therapies varies widely”. We acknowledge that this is a limitation in a survey across so diverse a selection of countries.*

Table 5 is unreadable.

***Response**: Please refer to the pdf version of the paper as this table has been distorted in the edited word document.*

On page 17, modelling of ENDS effects on health are only really focused on the US and other high income countries. It is unlikely that ENDS would have much impact in countries with very low smoking rates. Also the term ENDS is out of date and ought to be replaced with a broader term - nicotine vaping products (NVPs).

***Response**: We only included France, the UK and the US in our reporting of ENDS use as dispersion of ENDS in the other countries is currently too low to yield any meaningful numbers given our sample sizes.*

*We strongly agree that there is a need for harmonization regarding the terminology, nevertheless we suggest sticking to ENDS in this paper as this was term used in the survey.*

The data from the study ought to be made publicly available.

***Response**: Data are available under the terms of the Creative Commons Zero "No rights reserved" data waiver (CC0 1.0 Public domain dedication).*

*Open Science Framework: Global Poll 2018, https://doi.org/10.17605/OSF.IO/X4GQZ*

*These links are available at the end of the paper.*

While lots of interesting descriptive data are provide, the paper loses its value since the authors have not focused the presentation on any themes. I would advise reformatting the paper, and use a fewer number of tables to communicate a few key themes. Additional materials can be placed in supplemental tables/figures. I would also encourage the authors to use figures where possible since this would be easier for readers to follow.

***Response**: The objective of this paper was to give a very broad overview of quantitative results from the first Global Poll commissioned by the foundation. Therefore, we provided only descriptive analysis and did not perform any in-depth analysis by ways of more complex statistical models. We have decided to narrow down our focus in our next poll and are planning to publish more specific results from there.*

*We thank the reviewer for taking the time to thoroughly review this paper.*
